# vWCluster: Vector-valued optimal transport for network based clustering using multi-omics data in breast cancer

**DOI:** 10.1371/journal.pone.0265150

**Published:** 2022-03-14

**Authors:** Jiening Zhu, Jung Hun Oh, Joseph O. Deasy, Allen R. Tannenbaum

**Affiliations:** 1 Department of Applied Mathematics & Statistics, Stony Brook University, New York, NY, United States of America; 2 Department of Medical Physics, Memorial Sloan Kettering Cancer Center, New York, NY, United States of America; 3 Departments of Computer Science, Stony Brook University, New York, NY, United States of America; University of North Texas, UNITED STATES

## Abstract

In this paper, we present a network-based clustering method, called ***vector Wasserstein clustering*** (vWCluster), based on the vector-valued Wasserstein distance derived from optimal mass transport (OMT) theory. This approach allows for the natural integration of multi-layer representations of data in a given network from which one derives clusters via a hierarchical clustering approach. In this study, we applied the methodology to multi-omics data from the two largest breast cancer studies. The resultant clusters showed significantly different survival rates in Kaplan-Meier analysis in both datasets. CIBERSORT scores were compared among the identified clusters. Out of the 22 CIBERSORT immune cell types, 9 were commonly significantly different in both datasets, suggesting the difference of tumor immune microenvironment in the clusters. vWCluster can aggregate multi-omics data represented as a vectorial form in a network with multiple layers, taking into account the concordant effect of heterogeneous data, and further identify subgroups of tumors in terms of mortality.

## Introduction

Current large-scale cancer genome projects, such as The Cancer Genome Atlas (TCGA), provide a comprehensive molecular portrait of human cancers, including gene expression, copy number variation (CNV), and DNA methylation profiles. These offer unprecedented opportunities for exploring cancer biology that is characterized through various molecular functions and their complex interactions. Several computational methods for multi-omics data integration and further clustering have been proposed to identify tumor subgroups associated with distinct clinical outcomes, leveraging complementary information of multi-omics data [1]. iCluster uses a joint latent variable method across multi-omics types to model integrative clustering [2]. Recently, Alkhateeb *et al*. proposed a deep learning method to predict the 5-year interval survival of breast cancer based on multi-omics data integration [3]. Network based clustering methods using integrated multi-omics data have been proposed. Similarity network function (SNF) is a technique for combining multiple networks of each omics type into a single network, which is followed by a spectral clustering method to identify subtypes of tumors [4]. On the other hand, aWCluster utilizes a prior known network of gene products to integrate multi-omics data [5]. First, the data integration method yields an invariant measure for each node (gene). After repeating this process for each omics type, the invariant measures are integrated at each node. The Wasserstein distance, derived from optimal mass transport (OMT) theory [6, 7], is then computed between all pairs of samples on the network using the integrative invariant measure. The distance matrix is then input into a hierarchical clustering algorithm, resulting in clusters.

Representing data, e.g. as latent variables or weighted graphs, is essential to efficiently integrate multi-omics data while minimizing information loss. In this study, we propose a new method, called ***vector Wasserstein clustering*** (vWCluster), in which we employ a vector-valued version of the Wasserstein distance [8]. First, multi-omics data are represented as a multi-layer biological network, forming a layer for each single omics type. The Wasserstein distance is then computed on the vector-valued data in the network between all pairs of samples. The resulting distance matrix is then input into a hierarchical clustering method to identify subtypes of tumors. This method that represents multi-omics data vectorially on a network appears to be more straightforward to handle heterogeneous data compared to previously proposed methods while minimizing information loss.

The Wasserstein distance from OMT has increasingly received attention in data analysis due to its attractive property of (weak) continuity [6, 7]. In the present work, we will only use the $W_{1}$ version, also known as the *Earth Mover’s distance* (EMD). Other metrics commonly used on distributions, such as Kullback Leibler, Jensen-Shannon, or total variation, do not have the property [9, 10], which makes the metrics much more susceptible to the noise that is typically observed in medical data. Moreover, the Wasserstein distance is a metric for distributions defined on a metric space, which is essential for us to include the information from the weighted graphs used in this paper. Due to its attractive properties, OMT is becoming more and more widely used in signal processing, machine learning, computer vision, meteorology, statistical physics, quantum mechanics, and network theory [9, 11–15]. To even strengthen its power, several works deal with various extensions of the theory; see [11, 13, 16–18] and the references therein. In the present work, we empoly vector-valued extension of the Wasserstein distance [8, 19].

To the best of our knowledge, we are the first to use a vector-valued OMT methodology for the multi-omics data integration. We propose a general pipeline to analyze heterogeneous data in a multi-layer structure, employing a known protein-protein interaction network, and then cluster samples based on the resulting Wasserstein distance matrix. In the present work, our method is applied to multi-omics data from the two largest breast cancer studies: the Molecular Taxonomy of Breast Cancer International Consortium (METABRIC) and TCGA studies [20, 21]. In the following section, we describe the proposed method and data in detail.

## Background and methods

We developed a vector-valued OMT approach that integrates multi-omics data represented in a multi-layer network, on which we applied the $W_{1}$ Wasserstein distance (EMD). Accordingly, we will only outline the OMT theory in this special case. In the following, we first describe the basic concept of Wasserstein distance and then introduce the proposed method.

### Scalar-valued optimal transport

The $W_{1}$ Wasserstein distance (EMD) was first formulated by the French civil engineer and mathematician Gaspard Monge in 1781 [6, 7, 22, 23]. Originally, this subject was inspired by the problem of finding the optimal plan, relative to a given cost, for moving a pile of soil from a given location to another in a mass preserving manner. The original Monge’s formulation of OMT (in which the cost function is defined by the distance) may be given a modern expression as follows [6, 7]:

$$\begin{array}{c} W_{M}\left(\rho_{0},\rho_{1}\right)=\underset{T}{\text{inf}}\;\{\int_{S}||x-T\left(x\right)\left|\right|\rho_{0}\left(x\right)dx\;\left|\;T_{\#}\rho_{0}=\rho_{1}\right\}, \end{array}$$ (1)

where *S* denotes a subdomain of $R^{n}$, *T* is the transport map, and *ρ*_0_, *ρ*_1_ are two marginals. Here *T*_#_ denotes the push-forward of *T*. Therefore, the $W_{1}$ Wasserstein distance is the optimal cost with respect to the norm among all possible *T*.

As pioneered by Leonid Kantorovich [24], the Monge formulation of OMT may be relaxed by replacing transport maps *T* by couplings *π*:

$$\begin{array}{c} W_{K}\left(\rho_{0},\rho_{1}\right)=\underset{\pi \in \Pi (\rho_{0},\rho_{1})}{\text{inf}}\int_{S}||x-y||\pi \left(dx,dy\right), \end{array}$$ (2)

where Π(*ρ*_0_, *ρ*_1_) denotes the set of all the couplings between *ρ*_0_ and *ρ*_1_ (joint distributions whose two marginal distributions are *ρ*_0_ and *ρ*_1_). Despite the relaxation, one may show that Kantorovich and Monge formulations are equivalent in a number of cases under certain continuity constraints; see [6, 7] and the references therein.

One of the benefits of Eq (2) is that it amounts to a linear programming problem. Via duality theory, an equivalent form may be expressed as follows (see [22] for the proof):

$$\begin{array}{cc} & W_{\tilde{K}}\left(\rho_{0},\rho_{1}\right)=\underset{u}{\text{inf}}\int_{S}||u\left(x\right)\left|\right|dx \end{array}$$ (3a)

$$\begin{array}{cc} & {\text{div}}_{x}u\left(x\right)=\rho_{0}\left(x\right)-\rho_{1}\left(x\right), \end{array}$$ (3b)

where $u=\left(u_{1},u_{2},\ldots ,u_{n}\right):S\to R^{n}$ is the flux, and *div*_*x*_ denotes the divergence operator.

It is straightforward to extend Eq (3) to the discrete case by simply replacing the integral by an appropriate summation and replacing *div*_*x*_ by the discrete divergence operator:

$$\begin{array}{cc} & W_{G}(\rho_{0},\rho_{1})=\underset{u}{\text{min}}\sum_{i=1}^{|E_{G}|}\left|u_{i}\right| \end{array}$$ (4a)

$$\begin{array}{cc} & \rho_{0}-\rho_{1}-Du=0. \end{array}$$ (4b)

On the graph $G=(V_{G},E_{G})$, the fluxes *u*_*i*_ now are defined on the edges $E_{G}$, and $D\in R^{|V_{G}\left|\times \right|E_{G}|}$ denotes the incidence matrix of G with directionality, namely we need to specify the directions of the fluxes. Thus, in the matrix *D*, each column has two nonzero entries, where one is 1 whose row number is the starting point of an edge while the other nonzero entry is -1 whose row number is the ending point of that edge.

### Vector-valued optimal transport

A vector-valued density $\vec{\rho }={\left[\rho^{1}\left(x\right),\rho^{2}\left(x\right),\cdots ,\rho^{m}\left(x\right)\right]}^{T}$ on a given space *S* (continuous or discrete) may represent a physical entity that can mutate or transition between alternative manifestations, e.g., power reflected off a surface at different frequencies or polarizations. More formally, in the continuous setting, an *m*-layer *vector-valued density* $\vec{\rho }$ on $S\subseteq R^{n}$ is a map from *S* to $R_{+}^{m}$ whose total mass is defined as $\sum_{i=1}^{m}\int_{S}\rho^{i}\left(x\right)dx.$ As a distribution, we require its total mass to be 1. Note that the integral over *S* is just in general. If the space *S* is a discrete space, then the integral is replaced by summation.

Vector-valued optimal transport studies such distributions, which is of great theoretical and practical interest since it does not simply consider each layer separately, but explicitly models the relationships among layers [19]. A relationship is expressed as an additional graph structure that connects each layer. Specifically, each component of *ρ* is represented by a node of a graph $F=(V_{F},E_{F})$ and an edge between two nodes allows for direct transport between the corresponding layers. So $|V_{F}|=m$, which represents the cardinality of all the channels (layers), and $E_{F}$ is the set of all the direct connections between the layers.

Thus, the vector-valued optimal transport problem may be written as follows:

$$\begin{array}{cc} & W_{V}\left({\vec{\rho }}_{0},{\vec{\rho }}_{1}\right)=\underset{\vec{u},\vec{w}}{\text{inf}}\;\int_{S}\left|\right|\vec{u}\left(x\right)\left|\right|+\gamma ||\vec{w}\left(x\right)\left|\right|dx \end{array}$$ (5a)

$$\begin{array}{cc} & {\text{div}}_{x}\vec{u}\left(x\right)+{\text{div}}_{F}\vec{w}\left(x\right)={\vec{\rho }}_{0}\left(x\right)-{\vec{\rho }}_{1}\left(x\right), \end{array}$$ (5b)

where $\vec{u},\vec{w}$ are both vector-valued, *div*_*x*_ is the spatial divergence which is taken componentwise for each layer, and ${\text{div}}_{F}$ is the discrete divergence on the graph F which takes the flows between channels into account. Here *γ* ≥ 0 is a parameter to control flow between channels.

As in the scalar-valued case, we can extend the definition for distributions to a discrete graph G. The vector-valued formulation on a graph is then the following:

$$\begin{array}{cc} & W_{\tilde{V}}({\vec{\rho }}_{0},{\vec{\rho }}_{1})=\underset{u,w}{\text{min}}\sum_{i=1}^{|E_{G}|}\sum_{j=1}^{|V_{F}|}|u_{i}^{j}|+\gamma \sum_{i=1}^{|V_{G}|}\sum_{j=1}^{|E_{F}|}\left|w_{i}^{j}\right| \end{array}$$ (6a)

$$\begin{array}{cc} & {\vec{\rho }}_{0}-{\vec{\rho }}_{1}-D_{1}u-D_{2}w=0, \end{array}$$ (6b)

where *u* is the flux within each layer, *w* is the flux across layers, and *D*_1_ and *D*_2_ are two matrices of the discrete divergence operators for two graphs.

On the one hand, this is a generalized form of Eq (4) derived by replacing each original node in the graph G by another graph. On the other hand, this formulation may be understood as a distribution on a super-graph G×F. This super-graph is an irregular grid version of the Kronecker product. A slight difference from directly computing OMT distance on such a super-graph is that vector OMT on a graph here gives two different weights for the two different sets of edges. It is weighted vector-valued OMT. We later will see that two different kinds of fluxes via two graphs have different meanings.

### Multi-omics data from two breast cancer studies

Multi-omics data for METABRIC and TCGA breast cancer studies were downloaded from the cBioPortal database [25, 26]. The METABRIC dataset contains microarray gene expression of 24,368 genes from 1,904 samples and copy number variation (CNV) of 22,544 genes from 2,173 samples. The intersection of the two omics data resulted in 16,195 genes from 1,904 samples. The TCGA breast cancer dataset consists of RAN-Seq gene expression of 18,022 genes from 1,100 samples, CNV of 15,213 genes from 1,080 samples, and methylation of 15,585 genes from 741 samples. The intersection of the three omics data resulted in 7,737 genes from 726 samples.

vWCluster requires all the nodal values in the network to be positive, because of the Markov chain process (see Section Markov chain and stationary distribution). The only data preprocessing was to exponentiate CNV values to ensure their positive.

### Graph structures for analysis

We represented multi-omics data as vector-valued distributions on the gene (product) interaction network. The interaction network was derived from the Human Protein Reference Database (HPRD) [27]. The largest connected network component was found in the interaction of the HPRD and the gene list of METABRIC or TCGA breast cancer data, separately, resulting in 3,147 and 3,426 genes, respectively. As multi-omics data in the TCGA breast cancer cohort, gene expression, CNV, and methylation data were used, whereas in the METABRIC cohort, only gene expression and CNV data were available, thereby forming 3-vector and 2-vector distributions, respectively.

More specifically, the network for METABRIC consisted of two layers (gene expression and CNV), each of which had the same topology (the largest connected network component) derived from HPRD. The connection between the two layers was formed by connecting the two nodes for the same gene in each layer, yielding the graph F structure.

**Fig 1:**
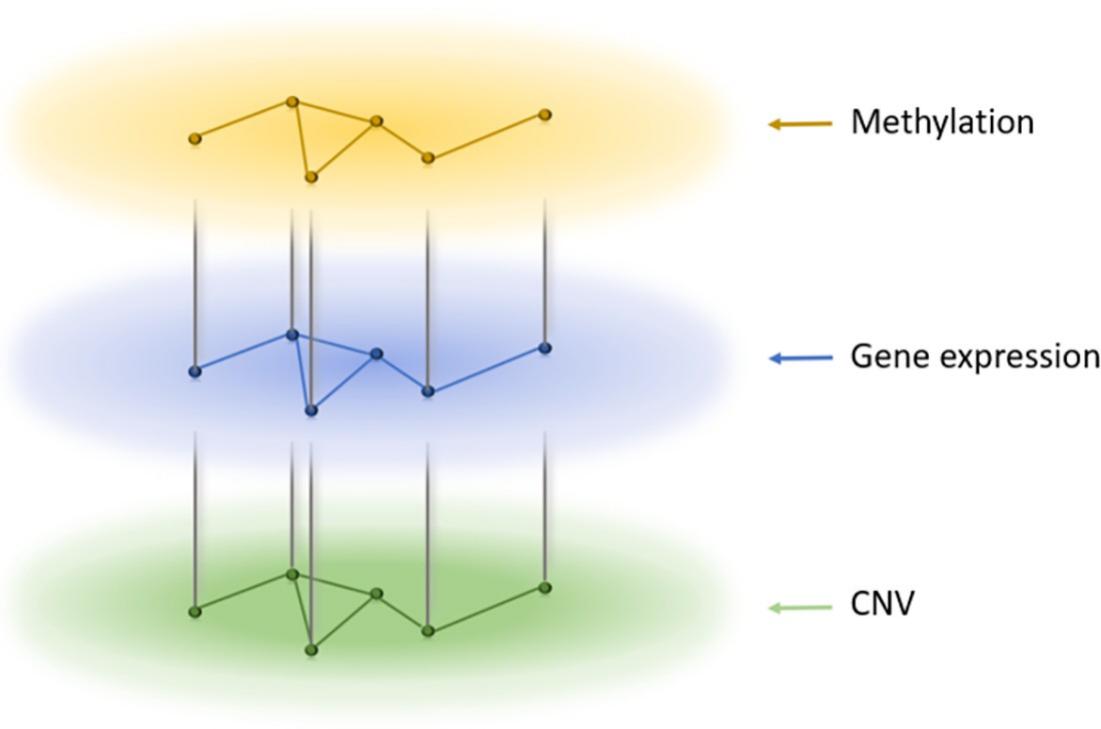
Graph structure for the TCGA breast cancer data.

For the network with the TCGA data, the layer for gene expression was connected with both layers for CNV and methylation since CNV and methylation may affect the level of gene expression. There was no connection between the CNV and methylation layers. See Fig 1.

### Markov chain and stationary distribution

One problem of applying the vector-valued optimal transport method to multi-omics data is that the scale of individual omics data varies. For example, CNV data consists of integer values, while gene expression and methylation data have continuous values. To tackle this issue, we use the invariant (stationary) distribution derived from a Markov process of the gene network.

A *Markov process* is a stochastic process such that the probability of a given event depends only on the state of the previous event. To put it simpler in our graph setting, one starts with a certain distribution. At each time step, the probability at each node redistributes to all its neighbors with predefined weights. In the gene network setting [28], we set the probability of moving from a node *i* to its neighbor *j* to be:

$$\begin{array}{c} p_{ij}=\frac{g_{j}}{\sum_{k\in N(i)}g_{k}}, \end{array}$$ (7)

where *g*_*k*_ > 0 is the weight of node *k*, which can be any omics type (gene expression, CNV or methylation). Note that for methylation, 1-methylation values were used since methylation is likely to be negatively correlated with gene expression.

The matrix *p* is a stochastic matrix, i.e., the state probability matrix from the current time step to the next, as follows:

$$\begin{array}{c} \pi^{t+1}=\pi^{t}p, \end{array}$$ (8)

where *π*^*t*^ is the distribution at time step *t*. In our setting, after a finite number of time steps, the initial distribution will converge to a stationary (invariant) distribution *π* such that

$$\begin{array}{c} \pi =\pi p. \end{array}$$ (9)

The stationary distribution has a closed form solution:

$$\begin{array}{c} \pi_{i}=\frac{1}{Z}g_{i}\sum_{k\in N(i)}g_{k}, \end{array}$$ (10)

where *Z* is the normalization factor to be a probability distribution.

This Markov process on the gene network mimics the interactions among genes and the stationary distribution gives a distribution that represents the information each gene has which includes not only its own value but the interactions with its neighbors. The Markov process was performed for each sample in individual omics types, separately, yielding invariant measures *I*_*ijk*_ for sample *i*, omics type *j*, and gene *k*.

### Clustering based on the vector-valued Wasserstein distance

With the graph structure determined, the vector-valued Wasserstein distance was computed for each pair of samples, using the invariant measures of each omics type. Note that the network for METABRIC or TCGA breast cancer data consisted of 2 and 3 layers, respectively. That is, we fitted the multi-omics data into the vector-valued optimal transport model. The resulting distance matrix was then input to standard hierarchical clustering to identify clusters of tumors. Kaplan-Meier survival analysis with log-rank test was performed to assess the difference of 5-year survival rates among the clusters identified. Further, CIBERSORT scores were compared among the clusters to investigate the difference in immune cell types [29, 30]. This analysis was performed for METABRIC or TCGA breast cancer data, separately. vWCluster was implemented in MATLAB and the code is available on https://github.com/MSK-MOI/vWCluster.

## Results

### METABRIC data analysis

**Fig 2:**
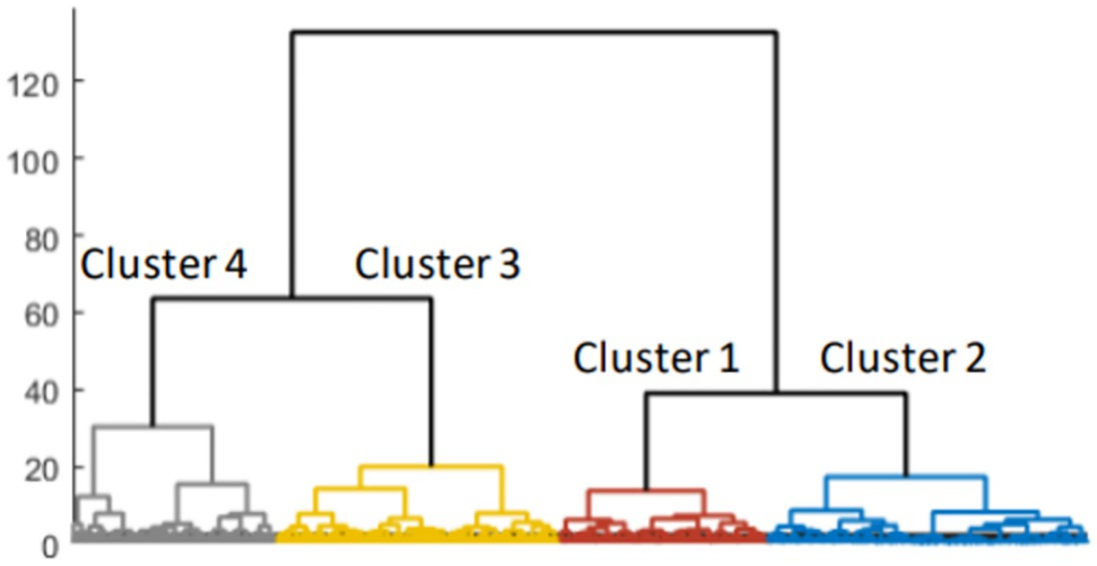
Clustering results employing the resultant vector-valued Wasserstein distance on METABRIC data.

The vector-valued Wasserstein distance was computed on gene expression and CNV data for METABRIC data. As described above, the resulting distance matrix was input to standard hierarchical clustering. The clustering results are shown in Fig 2.

**Fig 3:**
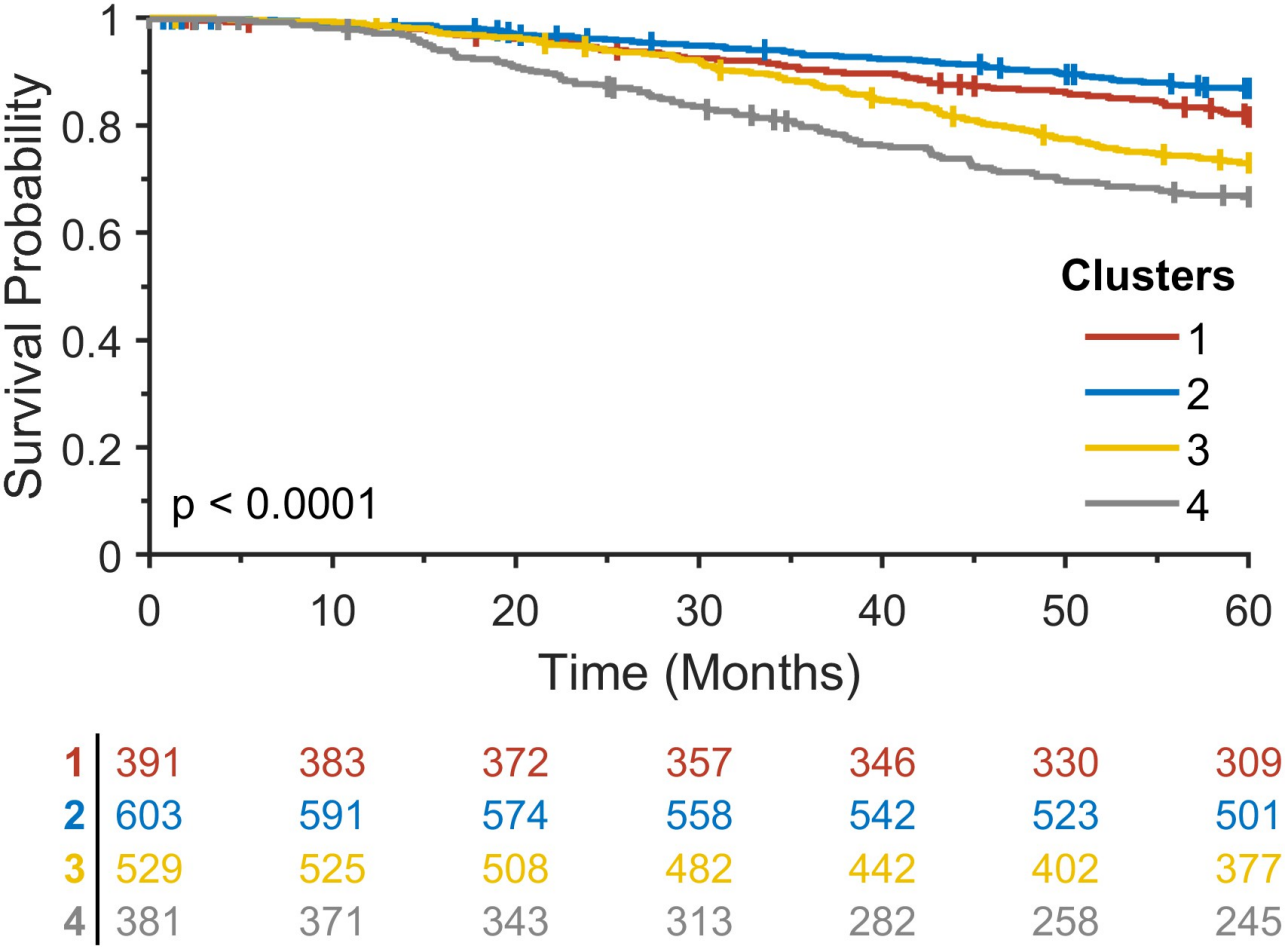
Kaplan-Meier analysis for four clusters that resulted from a hierarchical clustering method on the vector-valued Wasserstein distance matrix in the METABRIC study.

Based on the dendrogram and the number of intrinsic molecular subtypes in breast cancer, four clusters were chosen for further analysis. Kaplan-Meier analysis with log-rank test (without NA samples) resulted in a statistically significant survival difference among clusters with a log-rank p < 0.0001 (Fig 3).

The clustering results were compared with PAM50 and Claudin-low subtypes [31], and the associations were assessed using a chi-squared test, resulting in p < 0.0001 as shown in Table 1. Clusters 1 and 2 were enriched for Luminal A subtype. Cluster 3 was enriched for Luminal A and B subtypes, and cluster 4 was more enriched for basal subtype.

**Table 1:** Comparison between PAM50 along with Claudin-low subtypes and clusters identified by the proposed method.

Cluster	Luminal A	Luminal B	Her2	Basal	Claudin-low	Normal-like	NA
1	189	94	33	16	24	32	3
2	316	78	32	18	95	62	2
3	147	205	85	31	27	33	1
4	27	84	70	134	53	13	0

NA: not available.

For the four clusters identified, 22 CIBERSORT immune cell types were compared using one-way analysis of variance (ANOVA) test. The twelve immune cell types were statistically significantly different among the four clusters. The top two significant immune cell types were M0 and M1 macrophages, for which Cluster 4 had the highest values with mean = 0.158 (standard deviation [SD] = 0.094) and 0.103 (0.051), respectively (Table 2).

**Table 2:** Comparison of 22 CIBERSORT immune cell types among the four clusters identified in METABRIC, showing mean (standard deviation) values.

Immune cell types	Cluster 1	Cluster 2	Cluster 3	Cluster 4	P-value
B cells naive	0.008 (0.017)	0.008 (0.018)	0.008 (0.019)	0.007 (0.017)	0.8240
B cells memory	0.03 (0.036)	0.035 (0.047)	0.025 (0.031)	0.026 (0.031)	**3.60E-05**
Plasma cells	0.17 (0.098)	0.169 (0.097)	0.172 (0.093)	0.165 (0.089)	0.7405
T cells CD8	0.045 (0.05)	0.044 (0.048)	0.041 (0.049)	0.042 (0.047)	0.5181
T cells CD4 naive	0.014 (0.034)	0.017 (0.039)	0.013 (0.028)	0.009 (0.025)	**0.0028**
T cells CD4 memory resting	0.055 (0.06)	0.057 (0.058)	0.052 (0.057)	0.044 (0.053)	**0.0068**
T cells CD4 memory activated	0.001 (0.005)	0.001 (0.007)	0.001 (0.005)	0.001 (0.007)	0.6263
T cells follicular helper	0.057 (0.034)	0.053 (0.035)	0.055 (0.035)	0.068 (0.036)	**3.86E-10**
T cells regulatory (Tregs)	0.016 (0.02)	0.014 (0.02)	0.017 (0.021)	0.017 (0.021)	0.0771
T cells gamma delta	0.057 (0.045)	0.056 (0.044)	0.059 (0.044)	0.063 (0.044)	0.1604
NK cells resting	0.003 (0.013)	0.005 (0.016)	0.003 (0.011)	0.003 (0.013)	0.1057
NK cells activated	0.029 (0.026)	0.025 (0.026)	0.029 (0.025)	0.031 (0.028)	**0.0028**
Monocytes	0.017 (0.024)	0.021 (0.027)	0.015 (0.022)	0.019 (0.029)	**0.0018**
Macrophages M0	0.106 (0.095)	0.104 (0.092)	0.13 (0.098)	0.158 (0.094)	**3.50E-19**
Macrophages M1	0.076 (0.042)	0.069 (0.044)	0.081 (0.043)	0.103 (0.051)	**1.69E-28**
Macrophages M2	0.159 (0.089)	0.162 (0.095)	0.147 (0.077)	0.126 (0.067)	**6.74E-11**
Dendritic cells resting	0.003 (0.01)	0.005 (0.013)	0.003 (0.008)	0.005 (0.014)	**0.0323**
Dendritic cells activated	0.003 (0.01)	0.004 (0.013)	0.005 (0.017)	0.009 (0.023)	**3.89E-07**
Mast cells resting	0.147 (0.104)	0.148 (0.11)	0.144 (0.1)	0.101 (0.082)	**4.26E-13**
Mast cells activated	0.001 (0.004)	0.001 (0.005)	0.001 (0.004)	0.001 (0.008)	0.1828
Eosinophils	0 (0)	0 (0.001)	0 (0.001)	0 (0)	0.9508
Neutrophils	0.001 (0.005)	0.001 (0.004)	0.001 (0.006)	0.001 (0.002)	0.0684

### TCGA data analysis

**Fig 4:**
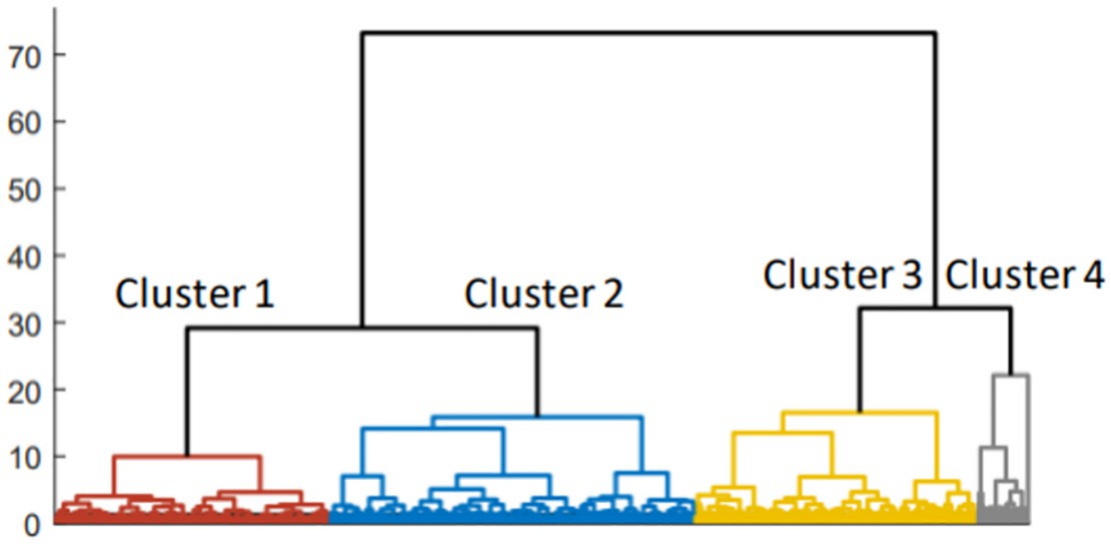
Clustering results employing the resultant vector-valued Wasserstein distance on TCGA data.

**Fig 5:**
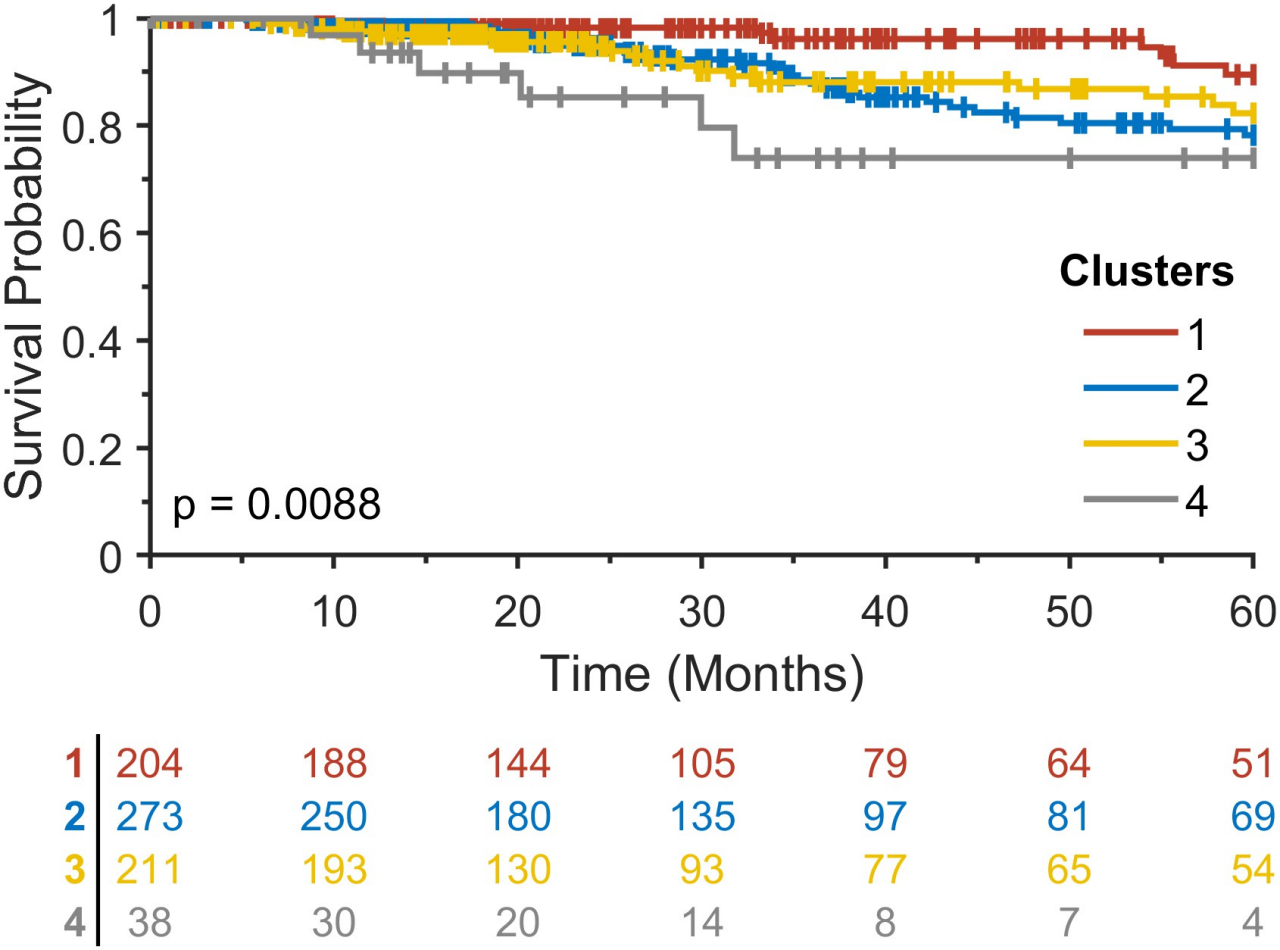
Kaplan-Meier analysis for four clusters that resulted from a hierarchical clustering method on the vector-valued Wasserstein distance matrix in the TCGA breast cancer study.

To validate the proposed method, we further analyzed multi-omics data in the TCGA breast cancer study, including gene expression, CNV, and methylation data. The clustering results are shown in Fig 4. Similar to the METABRIC analysis, four clusters were chosen for further analysis. Kaplan-Meier analysis with log-rank test resulted in a statistically significant survival difference among clusters with a log-rank p = 0.0088 (Fig 5).

The clustering results were compared with PAM50 subtypes. The associations were assessed using a chi-squared test, resulting in p < 0.0001 as shown in Table 3. Clusters 1 and 2 were enriched for Luminal A and B subtypes. Cluster 4 was more enriched for basal subtype.

**Table 3:** Comparison between PAM50 subtypes and clusters identified by the proposed method.

Cluster	Luminal A	Luminal B	Her2	Basal	Normal-like	NA
1	184	9	0	0	11	0
2	131	46	22	56	15	3
3	66	73	19	45	4	4
4	2	7	2	25	1	1

NA: not available.

For the four clusters identified, 22 CIBERSORT immune cell types were compared using the one-way ANOVA test. Fifteen immune cell types were statistically significantly different among the four clusters. The most significant immune cell type was M0 macrophages with p = 1.51E-08 (Table 4).

**Table 4:** Comparison of 22 CIBERSORT immune cell types among the four clusters identified in TCGA, showing mean (standard deviation) values.

Immune cell types	Cluster 1	Cluster 2	Cluster 3	Cluster 4	P-value
B cells naive	0.068 (0.045)	0.05 (0.046)	0.045 (0.046)	0.033 (0.037)	**2.29E-07**
B cells memory	0.007 (0.017)	0.015 (0.031)	0.01 (0.021)	0.017 (0.025)	**0.0005**
Plasma cells	0.049 (0.052)	0.037 (0.045)	0.046 (0.052)	0.032 (0.038)	**0.0341**
T cells CD8	0.106 (0.057)	0.108 (0.064)	0.093 (0.061)	0.094 (0.068)	**0.0331**
T cells CD4 naive	0 (0.004)	0 (0.001)	0.002 (0.011)	0 (0.002)	0.0662
T cells CD4 memory resting	0.135 (0.076)	0.122 (0.074)	0.098 (0.071)	0.068 (0.069)	**1.23E-06**
T cells CD4 memory activated	0 (0.002)	0.004 (0.012)	0.003 (0.011)	0.003 (0.009)	**0.0014**
T cells follicular helper	0.063 (0.039)	0.073 (0.04)	0.07 (0.045)	0.093 (0.068)	**0.0003**
T cells regulatory (Tregs)	0.014 (0.02)	0.026 (0.03)	0.02 (0.024)	0.015 (0.02)	**8.15E-06**
T cells gamma delta	0.003 (0.01)	0.003 (0.01)	0.002 (0.007)	0.004 (0.012)	0.4291
NK cells resting	0.003 (0.01)	0.006 (0.013)	0.005 (0.011)	0.012 (0.019)	**7.92E-05**
NK cells activated	0.02 (0.024)	0.019 (0.023)	0.021 (0.025)	0.017 (0.023)	0.8629
Monocytes	0.02 (0.023)	0.016 (0.019)	0.015 (0.026)	0.016 (0.018)	**0.0314**
Macrophages M0	0.056 (0.1)	0.087 (0.105)	0.116 (0.132)	0.177 (0.165)	**1.51E-08**
Macrophages M1	0.055 (0.032)	0.069 (0.045)	0.057 (0.04)	0.058 (0.054)	**0.0014**
Macrophages M2	0.271 (0.119)	0.273 (0.128)	0.301 (0.121)	0.286 (0.13)	0.1177
Dendritic cells resting	0.021 (0.031)	0.015 (0.025)	0.011 (0.024)	0.005 (0.012)	**0.0003**
Dendritic cells activated	0.002 (0.007)	0.004 (0.013)	0.009 (0.026)	0.021 (0.06)	**3.58E-07**
Mast cells resting	0.096 (0.071)	0.063 (0.062)	0.064 (0.075)	0.042 (0.044)	**1.41E-07**
Mast cells activated	0.007 (0.024)	0.005 (0.02)	0.011 (0.036)	0.004 (0.009)	0.4921
Eosinophils	0.001 (0.003)	0 (0.003)	0 (0.002)	0 (0)	0.8402
Neutrophils	0.003 (0.008)	0.002 (0.005)	0.004 (0.007)	0.003 (0.008)	0.1433

**Fig 6:**
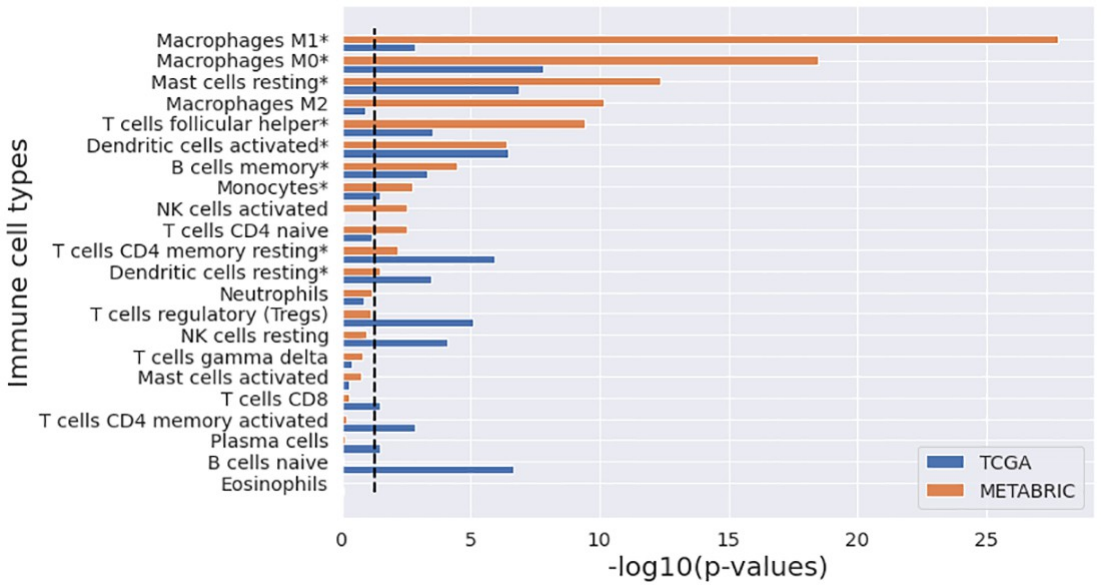
Comparison of 22 immune cell types in CIBERSORT among the four clusters identified in METABRIC and TCGA studies. The black dot line indicates -log10(p = 0.05).

Nine immune cell types were significantly different in both METABRIC and TCGA studies: memory B cells, resting memory CD4 T cells, follicular helper T cells, monocytes, M0 macrophages, M1 Macrophages, resting dendritic cells, activated dendritic cells, and resting mast cells (Fig 6). Three immune cell types, including naive CD4 T cells, activated NK cells, and M2 macrophages, showed statistical significance in METABRIC alone, whereas six immune cell types, including naive B cells, Plasma cells, CD8 T cells, activated memory CD4 T cells, regulatory T cells (Tregs), and resting NK cells, showed statistical significance in TCGA alone.

### Comparison with SNF

The clustering performance of vWCluster was compared with that of SNF using gene expression and CNV data for METABRIC and gene expression, CNV, and methylation data for TCGA breast cancer. Kaplan-Meier analysis for the resulting four clusters from SNF yielded statistical significance for METABRIC with a log-rank p < 0.0001, but for TCGA breast cancer, the log-rank test was statistically insignificant with p = 0.13 (S1 Fig).

We also compared vWCluster with SNF for five clusters. For METABRIC, both methods resulted in significant log-rank p-values with p < 0.0001. For TCGA breast cancer, vWCluster resulted in statistical significance with p = 0.013, which was slightly worse than that of four clusters, whereas the p-value of SNF for five clusters remained statistically insignificant with p = 0.0884.

## Discussion

The treatment of multi-omic biological data in a vector-valued manner may provide new insights for understanding the biological mechanisms of cancer biology, using complementary information offered by individual omics types. vWCluster is a data analysis methodology based on OMT theory, which enables the integration of multi-omics data in a vector-valued form, represented by multiple layers in a network. The Wasserstein distance computed on the vector-valued data was further employed to identify cancer subtypes. We applied this method to the two largest breast cancer studies, METABRIC and TCGA. The clusters identified showed significantly different survival rates in both studies.

vWCluster identified cluster 4 as a poor survival group in both METABRIC and TCGA breast cancer studies and cluster 2 and cluster 1 as a good survival group in METABRIC and TCGA breast cancer, respectively. In both studies, the poor survival group was enriched for basal subtype and the good survival group was enriched for Luminal A subtype. This is consistent with the clinical findings that in general, the triple-negative/basal-like subtype has a poor prognosis [32] while the Luminal A subtype has a better prognosis than other breast cancer subtypes [33].

CIBERSORT scores, consisting of 22 immune cell types, were further compared among the identified clusters. CIBERSORT employs gene expression profiles from a set of 547 genes to predict 22 immune cell types, using support vector regression [29]. ANOVA tests revealed that nine immune cell types were commonly statistically significant in both studies, indicating that the tumor immune microenvironment may differ among the identified clusters and this is associated with the difference in survival in breast cancer patients. Among the nine immune cell types, the poor survival group (cluster 4) had the lowest scores in memory resting CD4 T cells and resting mast cells, and the highest scores in follicular helper T cells, M0 macrophages, and activated dendritic cells in both METABRIC and TCGA breast cancer studies (Tables 2 and 4). By contrast, the good survival group (cluster 2 in METABRIC and cluster 1 in TCGA breast cancer) had the highest scores in memory resting CD4 T cells and resting mast cells, and the lowest scores in follicular helper T cells and M0 macrophages in both METABRIC and TCGA breast cancer studies. The score for activated dendritic cells was the lowest in TCGA breast cancer and the second lowest in METABRIC. A study revealed that M0 and M1 macrophages were significantly higher in the basal-like subtype compared to the Luminal A and B subtypes (p < 0.001) [34]. Recently, Gao *et al*. [35] investigated the difference of immune cells infiltration abundance between ER/PR-positive and triple-negative subtypes and reported that triple-negative tumors had significantly higher CIBERSORT scores for follicular helper T cells (p < 0.001) and lower CIBERSORT scores for resting memory CD4 T cells (p = 0.002) and resting mast cells (p < 0.001) compared to ER/PR-positive tumors. These results are consistent with our findings.

**Fig 7:**
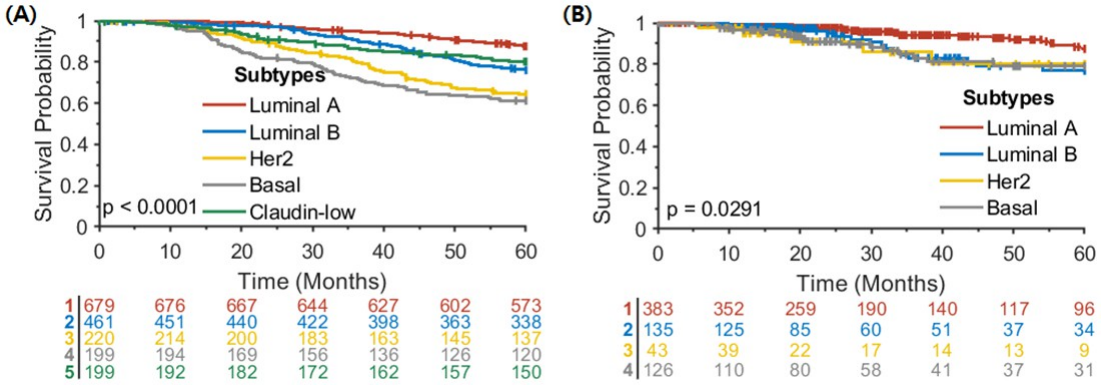
Kaplan-Meier analysis for intrinsic molecular subtypes in (A) METABRIC and (B) TCGA breast cancer studies with no normal-like samples.

Kaplan-Meier analysis was performed for intrinsic molecular subtypes in the METABRIC and TCGA breast cancer studies (Fig 7). As in Kaplan-Meier analysis for the clusters identified by our method in METABRIC, an extremely significant survival difference was found among intrinsic subtypes with a log-rank p < 0.0001, showing the worst survival rate for basal subtype. By contrast, for the TCGA breast cancer cohort, our method resulted in much better statistical significance with a log-rank p = 0.0088 compared to marginal statistical significance with a log-rank p = 0.0291 among intrinsic subtypes in TCGA. It is worth noting that Kaplan-Meier survival curves for all four clusters in Fig 5 were separable, whereas in the intrinsic subtypes, only the Luminal A subtype was separated from others that had similar survival patterns, suggesting the potential of our proposed method to identify new subtypes in cancer and further stratify patients at high risk of mortality. Further investigation of the association between the tumor immune microenvironment and survival will be explored in future work.

Prior to this study, Chen *et al*. [8] and Ryu *et al*. [19] introduced vector-valued extensions of the Wasserstein distance metric. However, the current study is the first to employ the vector-valued Wasserstein distance methodology for the integration of multi-omics data and further to cluster samples.

## Conclusion

We proposed a multi-omics data integration and clustering method, called vWCluster, based on the vector-valued Wasserstein distance. In this method, individual omics types represented as multiple layers in a network can be efficiently integrated, considering the biological interactions of biomarkers and providing complementary biological information. The formulation of vWCluster treats the data vectorially, which potentially minimizes information loss. vWCluster is flexible and applicable to the integration of multi-modal data including imaging and genomic data, which is a research direction we plan to explore in the future.

## Supporting information

S1 Fig Kaplan-Meier analysis for four clusters that resulted from SNF in (A) METABRIC and (B) TCGA breast cancer studies. (PDF)
