# Supplementary figures and images for: vWCluster: Vector-valued optimal transport for network based clustering using multi-omics data in breast cancer
Source: PLoS One. 2022 Mar 14;17(3):e0265150. doi: 10.1371/journal.pone.0265150 (PMC8920287; doi:10.1371/journal.pone.0265150)

(A)

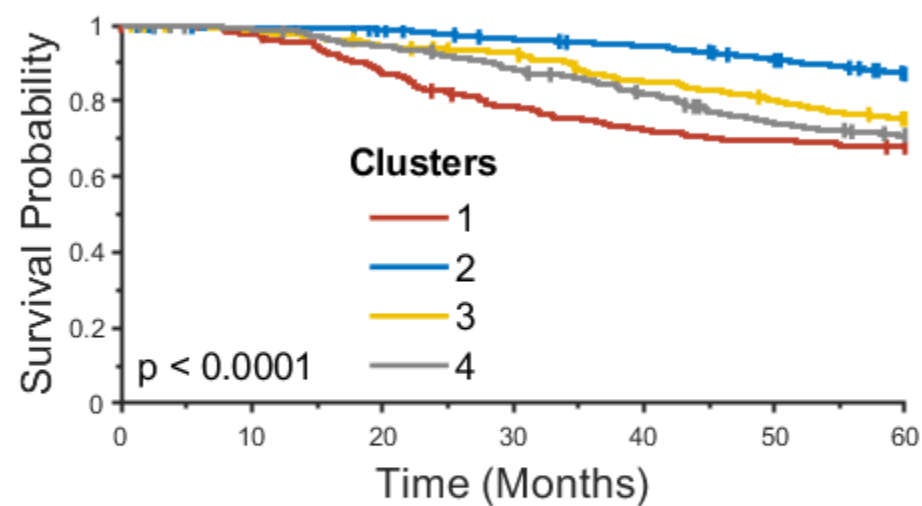

|   |     |     |     |     |     |     |     |
|---|-----|-----|-----|-----|-----|-----|-----|
| 1 | 214 | 208 | 185 | 165 | 151 | 146 | 141 |
| 2 | 776 | 765 | 756 | 733 | 717 | 686 | 650 |
| 3 | 359 | 351 | 336 | 326 | 297 | 279 | 260 |
| 4 | 555 | 546 | 520 | 486 | 447 | 402 | 381 |

(B)

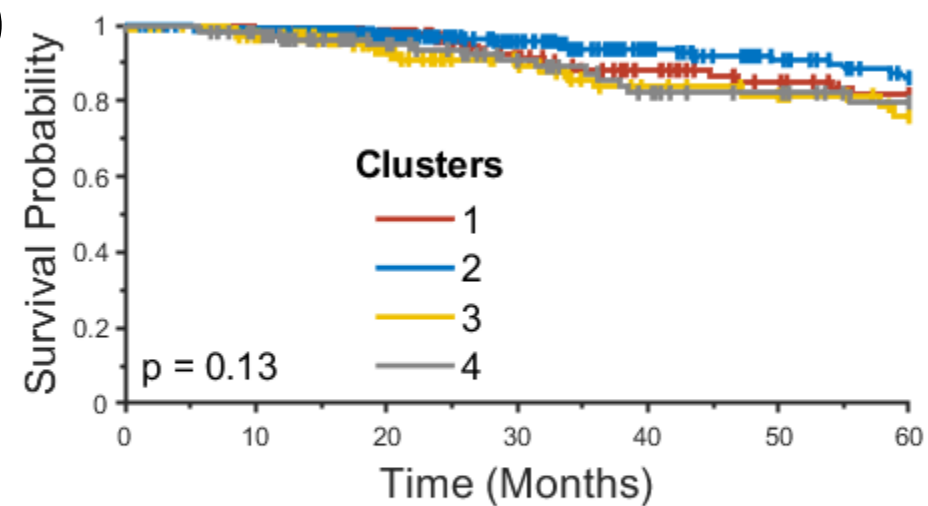

|   |     |     |     |     |     |    |    |
|---|-----|-----|-----|-----|-----|----|----|
| 1 | 187 | 176 | 118 | 84  | 67  | 55 | 48 |
| 2 | 313 | 286 | 210 | 151 | 109 | 87 | 70 |
| 3 | 115 | 98  | 71  | 54  | 39  | 35 | 27 |
| 4 | 111 | 101 | 75  | 58  | 46  | 40 | 33 |

Supplement: S1 Fig — (PDF) [file pone.0265150.s001.pdf]
